# Evaluation of an individual anamnesis tool for teaching risk-oriented prevention – a pilot study in undergraduate dental students

**DOI:** 10.1186/s12909-022-03710-2

**Published:** 2022-08-29

**Authors:** Gerhard Schmalz, Jacqueline Lange, Felix Krause, Rainer Haak, Bernd Lethaus, Rüdiger Zimmerer, Dirk Ziebolz

**Affiliations:** 1 Department of Cariology, Endodontology and Periodontology, University of Leipzig, Liebigstr. 12, 04103 Leipzig, Germany; 2 Clinic for Operative Dentistry, Periodontology and Preventive Dentistry, University Hospital RWTH Aachen, Aachen, Germany; 3 Department of Cranio Maxillofacial Surgery, University Clinic Leipzig, Leipzig, Germany

**Keywords:** Dental education, Risk classification, Medical history, Risk management

## Abstract

**Background:**

A comprehensive medical history is needed to establish and ensure a high standard in dental care; however, it is challenging to draw clinical consequences on the variety of potential diseases and medications, especially for dental students. Aim of this observational study was to investigate, whether undergraduate dental students using an analog anamnesis tool for risk classification would be more confident and have more knowledge in risk classification than other students in the same year of study.

**Methods:**

A cohort of 48 fifth year dental students was included and allocated into two groups based on their curriculum-related division (group A: *n* = 25, group B: *n* = 23). Group A received a teaching event and provision of an analog anamnesis tool for risk classification; group B received neither a teaching event nor the anamnesis tool. At baseline and after two weeks (follow-up), questionnaires regarding self-perceived confidence with risk classification, questions on different disease, medications and lifestyle factors and a task with 15 medical histories of prepared patient cases were applied. The data was statistically analyzed using Mann–Whitney or Wilcoxon test.

**Results:**

In group comparison of the differences between baseline and follow-up regarding self-perceived confidence, significantly higher improvement was noted in group A compared to group B for all questions (*p* < 0.05). With regard to knowledge, the group comparison revealed that the differences in all of the four tasks were significantly higher in group A compared to group B (pi ≤ 0.01). Thereby, the different tasks in group A differed between baseline and follow-up as follows: Risk of complications: 49.04 ± 13.59 vs. 67.96 ± 17.22, *p* < 0.01, Risk of oral diseases: 48.77 ± 13.57 vs. 63.44 ± 16.78, *p* = 0.01, Indication of antibiotic prophylaxis: 75.70 ± 13.45 vs. 87.97 ± 10.37, *p* < 0.01 and the Medical history task on 15 patient cases: 58.45 ± 4.74 vs. 71.47 ± 9.54, *p* < 0.01.

**Conclusion:**

The applied analog anamnesis tool supported an increase in students´ confidence with issues related to at-risk patients alongside with their knowledge in risk classification. The applied anamnesis tool can be recommended for improving teaching of risk management for undergraduate dental students.

## Background

To support safety, effectiveness and efficiency of dental preventive measures, the concept of individualized prevention, including a risk classification system to classify potential risks of complications and oral diseases has been introduced, recently [1]. Against the background of several diseases with the potential to cause complications during dental interventions, e.g. antithrombotic medication with related risk for bleeding complications, risk for infective endocarditis or medication-related osteonecrosis of the jaw [2–5], risk classification appears mandatory in dental practice. Moreover, the association between oral and systemic health has been repeatedly highlighted, whereby different general medical conditions, e.g. diabetes mellitus, rheumatic diseases or medications can increase the risk for development and/or progression of oral diseases [6–10]. Therefore, the detection and appropriate classification of respective risk factors in dental care appears of high relevance.

A previous study by this working group demonstrated that the risk classification system provided in the concept of individualized prevention was able to increase both, subjectively perceived and objectively measurable skills and knowledge in risk classification of undergraduate dental students [11]. This topic appears of increasing importance, as it is known that dental students are faced with a couple of different potential at-risk patients during their dental study [12]. Similarly, there is a necessity to foster students’ knowledge regarding risk factors for oral diseases, especially periodontitis [13]. While the need for teaching risk management in undergraduate dental education appears reasonable, the appropriate way of teaching remains unclear. One strategy could be the usage of problem-oriented learning [14], which has been combined with the risk classification system in the previous study [11]. Furthermore, experiential learning [15] or using standardized patients [16] were potential strategies. The major shortcoming of all of these approaches is the absence of a “tool” to allow a fast and safe risk classification of the patients.

Health history forms, which are used to gain information on general diseases, medications and lifestyle factors of patients are an important instrument in risk management and should ensure safe dental treatment [17]. An accurate medical history is needed to establish and ensure a high standard in dental care, especially regarding medication [18]. However, available forms are only questionnaires, including yes/no questions, without a direct link to the respective risks for the patients. Therefore, a comprehensive health record form was combined with the risk classification system for this current study. In a cohort of fifth year undergraduate dental students, this “anamnesis tool” should be tested regarding to its ability to increase subjective and objective knowledge of the participants. Accordingly, aim of this current study was to investigate, whether students using an analog anamnesis tool to classify the risk of patient cases would be subjectively more confident and have more knowledge on risk classification than other students in the same year of dental study. It was hypothesized that students using the anamnesis tool would be superior against the comparison group regarding their subjectively perceived and objectively measurable knowledge in risk classification.

## Methods

### Study design

This observational study with two weeks follow-up was reviewed and approved by the Ethics Committee of the medical faculty of Leipzig University, Germany (No. 487/20-ek). All participants were volunteer students, who were informed verbally and in writing about the study and gave a written informed consent for participation.

### Participants and groups

All participants (*n* = 48) were undergraduate dental students in their fifth year of dental studies. Based on the curriculum of Leipzig University, the students of one study year are divided in two groups, of which one group undergoes the clinical course in conservative dentistry and periodontology, while the other group undergoes the clinical course in prosthodontics. Based on that group allocation in the winter term 2020/2021, group A (conservative dentistry and periodontology, teaching event and provision of an analog anamnesis tool), and group B (prosthodontics, neither a teaching event nor using the anamnesis tool) were recruited.

### Risk classification system and anamnesis tool

The risk classification system was described and introduced previously [1, 11]. In brief, each risk factor of a patient, including systemic diseases, medication and lifestyle is categorized into a low, moderate or high risk of complications and/or oral diseases. Thereby, a risk of complications means that there is increased probability of harm of the patient during dental intervention. A risk of oral diseases describes the increased likelihood of development or progression of an oral disease related to the respective risk factor [1]. On this basis, an analog anamnesis tool was developed to support fast and safe risk classification of a patient case. Therefore, a comprehensive medical history form was combined with the applied risk classification system. For example, the question “Do you have diabetes mellitus” was classified as follows: no = low risk of complications and low risk of oral diseases, yes, HbA1c < 7% = moderate risk of complications and moderate risk of oral diseases, yes, HbA1c > 7% = high risk of complications and high risk of oral diseases [19, 20]. The question “Do you have a heart valve replacement” was classified as no = low risk of complications and low risk of oral diseases, yes = high risk of complications (antibiotic prophylaxis needed) and low risk of oral diseases [3]. This principle was applied for all questions of the medical history form to generate the anamnesis tool. For visualization of the respective risks, colors according to a traffic-light system were applied, where the three colors represented the respective risk, i.e. green = low, yellow = moderate and red = high risk.

### Teaching of the risk classification system and anamnesis tool

Group A received a teaching event regarding risk classification and the anamnesis tool (60 min). Therefore, the risk classification system as well as the anamnesis tool were introduced in a presentation of twenty minutes. After this, small groups of 3–4 students were randomly built by the drawing of lots, and these small groups applied the anamnesis tool on patient cases provided by the teacher. In the last third of the teaching event, students presented and discussed their results of small group work together in the plenum.

### Questionnaires

A general questionnaire was applied to assess age, gender and any medical occupation prior to dental study. To evaluate the knowledge of the students, different questionnaires and tasks were applied. First, to assess the ability to classify patient cases, 15 medical histories of prepared patient cases were used. For each of these cases, the risk of complications (low, moderate or high) and the risk of oral diseases (low, moderate or high) was asked with regard to systemic diseases, medication and lifestyle of the patient case (i.e. six answers each patient case, in total 90 answers). Furthermore, each 15 risk factors (8 systemic diseases, 5 medications, and 2 lifestyle factors) were asked regarding a potential risk of complications, risk of oral diseases as well as the necessity of an antibiotic prophylaxis.

To evaluate the self-perceived confidence with risk classification, five questions were asked on a 10-point Likert scale (1 = very low, 10 = very high). The questionnaires were composed in line with the previous teaching study on risk-oriented prevention [11]. The previously designed questionnaires were modified for the current study and answered independently by five students, which were not included in the current study, to validate the understandability of the questionnaires and to make corrections, if necessary. Moreover, a group of three dentists and three dental students checked all the answers of knowledge questions and ensured that there was always only one clearly correct answer. This answer was finally used as reference to evaluate the answers of the study participants as “correct” or “incorrect”.

### Study flow

**Fig. 1:**
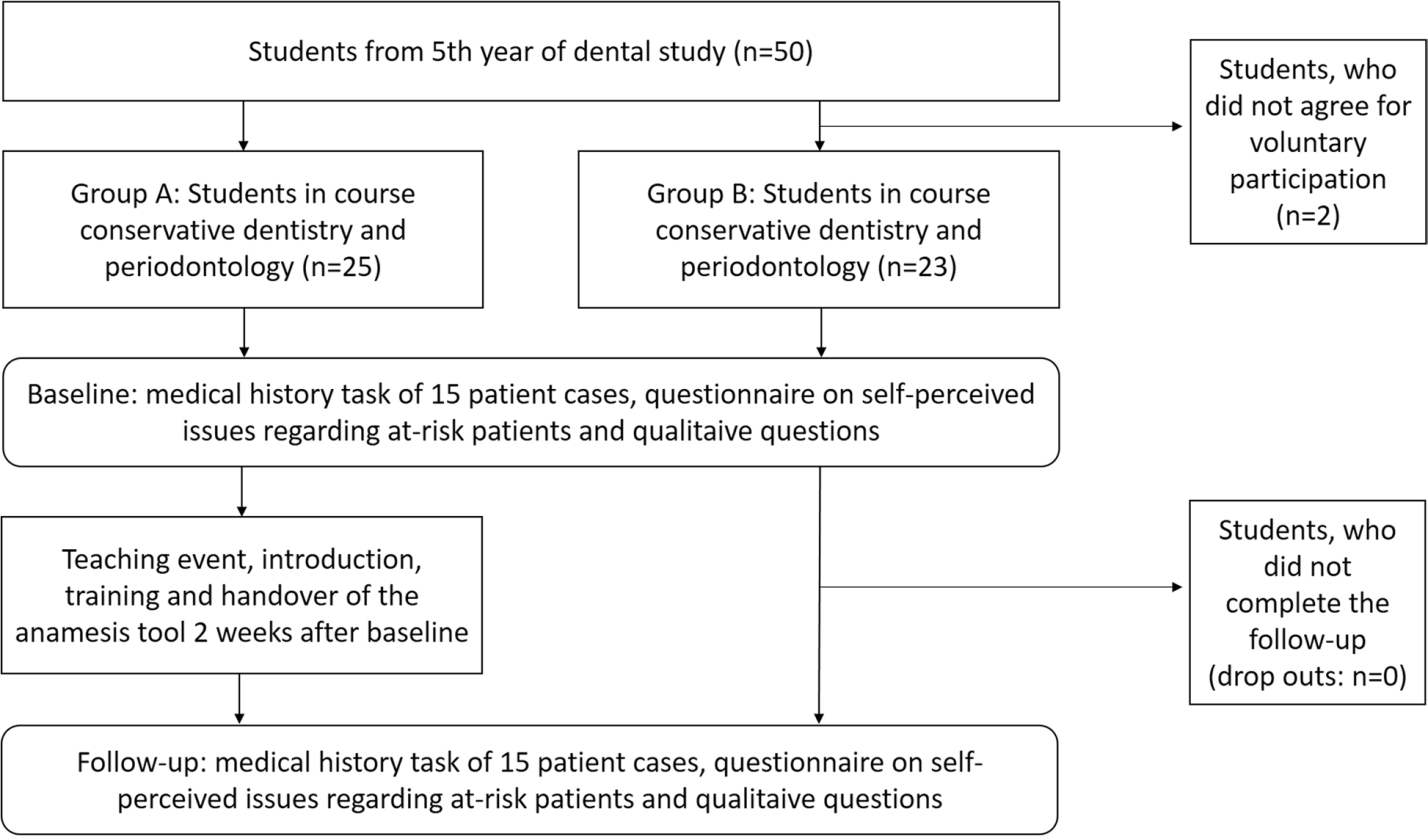
Study flow

An overview on the study flow is given in Fig. 1. All students were asked for their voluntary participation and, in case of written informed consent, included in the current study. At baseline, all students received the questionnaires as described above. After two weeks, Group A received the teaching event, while no form of teaching risk classification or provision of the anamnesis tool was given to group B. After this, Group A received the task of 15 medical histories alongside with the anamnesis tool, as well as the further questionnaires. Group B received the same questionnaires, but needed to solve them without using the anamnesis tool. The 15 patient cases as well as 15 risk factors were equal at baseline and follow-up for both groups to ensure the same level of difficulty.

### Statistical analysis

The statistical analysis was performed using SPSS for Windows, Version 24.0 (SPSS Inc., U.S.A.). Metric variables were tested for their normal distribution with Shapiro–Wilk-test, showing a non-normal distribution for the vast majority of parameters (*p* < 0.05). Comparing two independent, non-normal distributed samples, Mann–Whitney-U-test was applied. Categorical or nominal data were analyzed by Wilcoxon test, respectively. Significance level was set at *p* < 0.05, whereby two-sided significance testing was executed for all tests.

## Results

### Participants

In group A, 25 students were included, while in group B 23 students could be enrolled. All participants completed the follow-up (Fig. 1). The age and gender distribution was comparable between the two groups (Table 1).

**Table 1:** Characteristics of participants. Values are given as absolute numbers, mean value ± standard deviation [median], or percentage. Significance level: *p* < 0.05

	**Group A**	**Group B**	***p*-value**
Number of participants baseline	25	23	-
Number of participants follow-up	25	23	-
Age	23.68 ± 1.76 [23]	24.09 ± 2.39 [23]	0.78
Gender male	20%	22%	0.67
Medical occupation before dental study	12%	13%	0.88

### Subjectively experienced issues regarding risk classification and identification of at-risk patients

#### Comparison within groups (Table 2)

**Table 2:** Subjectively experienced issues within groups regarding at-risk patients in dental prevention at baseline and follow-up (after 2 weeks), values are given as mean values ± standard deviation [median]; 1 = not at all, 10 = very good/very important. Significant values (*p* < 0.05, Wilcoxon test) are highlighted in bold

	**Group A**			**Group B**		
	**Baseline**	**Follow-up**	***p*** **-value**	**Baseline**	**Follow-up**	***p*** **-value**
**How confident are you with general diseases?**	5.16 ± 1.72 [5]	5.96 ± 1.65 [6]	0.11	5.48 ± 1.50 [5]	4.87 ± 1.55 [5]	**0.04**
**How confident are you with medication?**	3.58 ± 1.51 [3]	4.60 ± 1.78 [4]	**0.01**	4.61 ± 1.75 [4]	4.52 ± 1.53 [4]	0.92
**How confident are you with lifestyle factors?**	6.48 ± 1.69 [6]	7.52 ± 1.69 [8]	**0.04**	6.91 ± 1.35 [7]	6.04 ± 1.46 [6]	**0.02**
**How good is your knowledge about at-risk patients?**	5.08 ± 1.50 [5]	5.64 ± 1.44 [6]	0.08	5.57 ± 1.12 [6]	5.04 ± 1.69 [5]	0.09
**How important is the identification of at-risk patients for you?**	9.16 ± 1.18 [9]	9.56 ± 0.87 [10]	**0.05**	9.35 ± 0.71 [9]	8.65 ± 1.37 [9]	**0.01**

Within group A, an increase in students’ ratings between baseline and follow-up was found for the following questions: “How confident are you with medication?” (*p* = 0.01), “How confident are you with lifestyle factors?” (*p* = 0.04) and “How important is the identification of at-risk patients for you?” (*p* = 0.05). Within group B, ratings for the following questions were significantly different between baseline and follow-up: “How confident are you with general diseases?” (*p* = 0.04), “How confident are you with lifestyle factors?” (*p* = 0.02) and “How important is the identification of at-risk patients for you?” (*p* = 0.01) (Table 2).

#### Group comparison (Table 3)

**Table 3:** Group comparison of the differences (baseline to follow-up) between the two groups with regard to the subjectively perceived issues on at-risk patients. Values are given as mean values ± standard deviation [median]. Significant values (*p* < 0.05, Mann–Whitney-U-test) are highlighted in bold

	**Difference baseline-follow-up**		
	**Group A**	**Group B**	***p*** **-value**
**How confident are you with general diseases?**	0.80 ± 2.18 [0]	-0.61 ± 1.34 [0]	**0.04**
**How confident are you with medication?**	1.02 ± 1.69 [1]	-0.09 ± 1.41 [0]	**0.02**
**How confident are you with lifestyle factors?**	1.04 ± 2.26 [1]	-0.87 ± 1.71 [-1]	**< 0.01**
**How good is your knowledge about at-risk patients?**	0.56 ± 1.50 [0]	-0.52 ± 1.41 [0]	**0.02**
**How important is the identification of at-risk patients for you?**	0.40 ± 0.91 [0]	-0.70 ± 1.40 [0]	**< 0.01**

In comparison between the groups A and B regarding their differences in students´ ratings of the five questions on subjectively experienced issues between baseline and follow-up, significantly higher improvement was noted in group A compared to group B for all questions (*p* < 0.05, Table 3).

### Assessment of students’ knowledge (knowledge questions)

#### Comparison within groups (Table 4)

**Table 4:** Percentage of correct answers of knowledge questions within the two groups at baseline and follow**-**up (after 2 weeks). Values are given as mean values ± standard deviation [median]. Significant results (*p* < 0.05, Wilcoxon test) are highlighted in bold

	**Group A**			**Group B**		
	**Baseline**	**Follow-up**	***p*** **-value**	**Baseline**	**Follow-up**	***p*** **-value**
**Risk of complications**	49.04 ± 13.59 [46.6]	67.96 ± 17.22 [66.6]	**< 0.01**	47.66 ± 9.40 [46.6]	41.52 ± 8.66 [41]	**0.01**
**Risk of oral diseases**	48.77 ± 13.57 [46.6]	63.44 ± 16.78 [66.6]	**0.01**	43.43 ± 13.88 [41]	45.89 ± 9.02 [46.6]	0.31
**Indication of antibiotic prophylaxis**	75.70 ± 13.45 [80]	87.97 ± 10.37 [93.3]	**< 0.01**	74.98 ± 10.61 [80]	75.49 ± 6.33 [76.43]	0.84
**Medical history task on 15 patient cases**	58.45 ± 4.74 [56.67]	71.47 ± 9.54 [74.45]	**< 0.01**	55.13 ± 4.47 [54.54]	54.35 ± 4.89 [54.33]	0.44

Within group A, the percentage of correct answers at follow-up compared to baseline was higher in all tasks, i.e. Risk of complications (*p* < 0.01), Risk of oral diseases (*p* = 0.01), Indication of antibiotic prophylaxis (*p* < 0.01) and the Medical history task on 15 patient cases (*p* < 0.01). Within group B, a difference between baseline and follow-up was only revealed for classification of the Risk of complications (*p* = 0.01), while the three other tasks were comparable between baseline and follow-up (*p* > 0.05, Table 4).

#### Group comparison (Table 5)

**Table 5:** Group comparison of the differences (baseline to follow-up) between the two groups with regard to the knowledge questions, i.e. percentage of correct answers. Values are given as mean values ± standard deviation [median]. Significant values (*p* < 0.05, Mann–Whitney-U test) are highlighted in bold

	**Difference baseline-follow-up**		
	**Group A**	**Group B**	***p*** **-value**
**Risk of complications**	18.92 ± 22.83 [20]	-6.15 ± 11.39 [-9.23]	**< 0.01**
**Risk of oral diseases**	14.67 ± 21.60 [13.4]	2.46 ± 15.76 [0]	**0.01**
**Indication of antibiotic prophylaxis**	12.27 ± 19.22 [11.2]	0.53 ± 10.48 [0]	**< 0.01**
**Medical history task on 15 patient cases**	13.02 ± 9.26 [14.45]	-0.78 ± 6.35 [-0.82]	**< 0.01**

Comparing the groups A and B, the differences in all of the four tasks was significantly higher in group A compared to group B (p_i_ ≤ 0.01, Table 5).

## Discussion

### Main results

The students, who underwent the teaching event and received the analog anamnesis tool felt more confident with issues related to at-risk patients than the comparison group. Moreover, these students had a higher improvement in correct answers of the different risk classification tasks compared to the control group.

### Comparison with available literature

This is the first teaching study of an individually developed analog anamnesis tool for risk classification in dental education. If compared to the previous study, which examined the risk classification system without a specific tool, a higher positive effect was observed in the current study; thereby, all of the tasks were answered with a remarkably higher amount of correct answers, including risk of complications (previous study: 7% vs. current study: 18.9%), risk of oral diseases (8% vs. 14.7%) and indication for antibiotic prophylaxis (2% vs. 12.3%) [11]. This comparison is limited by the fact that the previous study used other tasks and less questions. Moreover, the medical history task of 15 patient cases was exclusively performed in the current study. Regardless, the provision of the anamnesis tool seems to bring more benefit than the risk classification system alone.

Medical problems are common in patients attending dental treatment in educational institutions; a German study evaluated that patients in dental student courses had in median one internal disease, disorder or syndrome, which was dominated by cardiovascular diseases, diabetes and pulmonal diseases [12]. Another study in the West Indies showed that 42 percent of patients attending a dental school had at least one medical condition [21]. Beside of the detection of these potential risk factors, their appropriate interpretation and derivation of a clinical consequence is an important issue. This is a major challenge in dental education, whereby different approaches have been discussed previously, including one-day course on oral anticoagulation [22], self-reflection [23], case-based learning [24], standardized patients [16] or experimental learning [15], to foster interprofessional education and competencies to solve challenges related to risk management. Although these approaches are related to a certain benefit of students, there is no practical tool available to support risk management in education and dental practice. Especially the assessment and interpretation of a medical history appears a promising approach, especially for dental practice; it has been described that often only a transcription of information to the dental chart is given in dental education, whereby the link to a practical consequence is missing [25]. This is the entry point of the anamnesis tool in the current study, which combined medical history with the concept of risk-oriented individual prevention [1]. Nearly 30 years ago, recommendations to improve the content and format of medical history forms in dental education were formulated, which also include the practical consequence related to the form [17]. Furthermore, it has been reported that students overestimate their performance in medical history, underlining a need for standardized criteria in medical history record and interpretation [26]. The anamnesis tool in the current study appears to fulfil this demand by significantly improving the performance of the students.

Several observations in the current study need further discussion. In group B (comparison group), a deterioration of correct answers was obvious, what appears conspicuous. This was similar in the previous study [11]. A potential explanation could be a weariness or frustration in the comparison group to answer the large number of questions; however, the same reason could apply to group A, which had exactly the same number and content of questions, while their scores improved significantly. Therefore, the number and composition of questions appears no reasonable explanation. Nevertheless, participants in group B might be less motivated to answer the questions correctly, because they did not have any intervention between baseline and follow-up. Regardless, the results appear to be explained by a missing (perceived) effect in this group rather than by their “weariness to answer”. A feedback, e.g., with open-ended questions after the second evaluation would have a certain potential to reveal an explanation for the negative effect in group B. While the current study did not consider this fact, because this result was not expected previously, further evaluations should consider this issue. The largest difference between groups A and B was in risk of complications and necessity of an antibiotic prophylaxis (see Table 5). Especially the correct use of an antibiotic prophylaxis is a very important issue of highest clinical relevance [27]. A Saudi-Arabian study found that 50% of students lacked knowledge regarding the medical conditions requiring antibiotic prophylaxis [28]. This is comparable to the baseline values in the current study. With the anamnesis tool, a significant improvement was achieved, but the value is still far below 100%, underlining that further interventions would be needed to foster this issue. Furthermore, the students perceived an increased confidence with issues regarding risk classification, but this effect was quite small, even if it was significant. This might also indicate that students need more support and continuous training to feel deeply familiar with risk classification and management.

### Strengths and limitations

This is the first study on an individual analog anamnesis tool for teaching risk-oriented prevention in undergraduate dental students. The inclusion of more than 20 students each group and the application of comprehensive tasks to evaluate the gain in confidence as well as objectively measurable knowledge in risk classification strengthen the current study´s findings. However, several limitations need to be recognized. Only fifth year students within one term were recruited and the follow-up was only two weeks. Accordingly, students from the earlier dental study years and prolonged periods, with or without consecutive training should be considered for further evaluation of the anamnesis tool. Moreover, there was no power calculation performed and therefore it is unclear whether the 25 or 23 students each group were enough participants to ensure statistically robust conclusions. Students within both groups were in the same year of studies and at the same faculty, making a strict separation of both groups impossible. However, on the one hand, recruiting students from the same faculty was necessary to ensure comparability of the groups. On the other hand, members of group A and B underwent different clinical courses and were therefore separated in the study period; additionally, group A was not allowed to talk about the anamnesis tool with members of group B. Of course, there was no possibility to check this during the follow-up, making it a limitation of the current study. Neither the questionnaires nor the anamnesis tasks were evaluated previously; while no standardized or established questionnaires or tasks are available, these self-composed questionnaires were applied. Thereby, their structure aligned to the previous study [11], but was extended to target a comprehensive insight into the knowledge of participants. Questionnaires, which measure latent variables require a complete study of validation, for example using the structural equation modeling; this was not performed in the current pilot study prior to application of the questionnaire, what limits the conclusions and should be recognized in future studies. Additionally, the study design does not allow assessing the positive effect of the anamnesis tool alone, because it was combined with the teaching of risk classification system; to clearly detect the isolated effect of the tool, a comparison of two groups receiving a teaching event, of which one group additionally gets the anamnesis tool, would be recommendable as a subsequent project. For orientation, a comparison to the previous study was discussed, showing a higher improvement in the anamnesis tool users within the current study [11]. Altogether, the current study provides some interesting results and must be seen as a pilot study, whereby the anamnesis tool needs further evaluation.

## Conclusion

Within the limitations of this pilot study, the applied anamnesis tool helped students to increase their confidence with issues related to at-risk patients alongside with their knowledge in risk classification. Accordingly, the anamnesis tool can be recommended to support teaching in risk management for undergraduate dental students.

## Data Availability

The datasets generated during and analyzed during the current study are not publicly available due to [pseudonymisation procedure according to the ethics vote] but are available from the corresponding author on reasonable request.

## References

[1] Schmalz G, Ziebolz D (2020). Changing the focus to the whole patient case instead of one oral disease - the concept of individualized prevention. Adv Prev Med.

[2] Felix J, Chaban P, Ouanounou A (2020). Dental management of patients undergoing antithrombotic therapy. J Can Dent Assoc.

[3] Wilson W, Taubert KA, Gewitz M (2007). Prevention of infective endocarditis. Guidelines from the american heart association. A guideline from the american heart association rheumatic fever, endocarditis and kawasaki disease committee, council on cardiovascular disease in the young, and the council on clinical cardiology, council on cardiovascular surgery and anesthesia, and the quality of care and outcomes research interdisciplinary working group. Circulation.

[4] Costantinides F, Clozza E, Ottaviani G, Gobbo M, Tirelli G, Biasotto M (2014). Antibiotic prophylaxis of infective endocarditis in dentistry: clinical approach and controversies. Oral Health Prev Dent.

[5] Beth-Tasdogan NH, Mayer B, Hussein H, Zolk O (2017). Interventions for managing medication-related osteonecrosis of the jaw. Cochrane Database Syst Rev.

[6] Dörfer C, Benz C, Aida J, Campard G (2017). The relationship of oral health with general health and NCDs: a brief review. Int Dent J.

[7] Winning L, Linden GJ (2017). periodontitis and systemic disease: association or causality?. Curr Oral Health Rep.

[8] Lalla E, Papapanou PN (2011). Diabetes mellitus and periodontitis: a tale of two common interrelated diseases. Nat Rev Endocrinol.

[9] Kaur S, White S, Bartold PM (2013). Periodontal disease and rheumatoid arthritis: a systematic re-view. J Dent Res.

[10] Potempa J, Mydel P, Koziel J (2017). The case for periodontitis in the pathogenesis of rheumatoid arthritis. Nat Rev Rheumatol.

[11] Schmalz G, Krause F, Grzelkowski M, Merle C, Rotzoll D, Haak R (2020). Evaluation of an educational concept for risk-oriented prevention in undergraduate dental education. BMC Med Educ.

[12] Humbert A, Schmage P, Harendza S (2018). Internal diseases encountered by dental students while treating dental patients during undergraduate training. BMC Med Educ.

[13] Friesen LR, Walker MP, Kisling RE, Liu Y, Williams KB (2014). Knowledge of risk factors and the periodontal disease-systemic link in dental students' clinical decisions. J Dent Educ.

[14] Tack CJ, Plasschaert AJ (2006). Student evaluation of a problem-oriented module of clinical medicine within a revised dental curriculum. Eur J Dent Educ.

[15] Watters AL, Stabulas-Savage J, Toppin JD, Janal MN, Robbins MR (2015). Incorporating experiential learning techniques to improve self-efficacy in clinical special care dentistry education. J Dent Educ.

[16] Anders PL, Scherer YK, Hatton M, Antonson D, Austin-Ketch T, Campbell-Heider N (2016). Using standardized patients to teach interprofessional competencies to dental students. J Dent Educ.

[17] Minden NJ, Fast TB (1994). Evaluation of health history forms used in U.S. dental schools. Oral Surg Oral Med Oral Pathol.

[18] Fitzgerald J, Epstein JB, Donaldson M, Schwartz G, Jones C, Fung K (2015). Outpatient medication use and implications for dental care: guidance for contemporary dental practice. J Can Dent Assoc.

[19] Carey IM, Critchley JA, DeWilde S, Harris T, Hosking FJ, Cook DG (2018). Risk of infection in type 1 and type 2 diabetes compared with the general population: a matched cohort study. Diabetes Care.

[20] Papapanou PN, Sanz M, Buduneli N, Dietrich T, Feres M, Fine DH (2018). Periodontitis: consensus report of workgroup 2 of the 2017 world workshop on the classification of periodontal and peri-implant diseases and conditions. J Clin Periodontol.

[21] Al-Bayaty HF, Murti PR, Naidu RS, Matthews R, Simeon D (2009). Medical problems among dental patients at the school of dentistry, the university of the west Indies. J Dent Educ.

[22] Martínez-Beneyto Y, López-Jornet P, Camacho-Alonso F, González-Escribano M (2012). Dental students’ knowledge of and attitudes toward anticoagulation dental treatment: assessment of a one-day course at the University of Murcia. Spain J Dent Educ.

[23] Kanthan R, Senger JL (2011). An appraisal of students’ awareness of “self-reflection” in a first-year pathology course of undergraduate medical/dental education. BMC Med Educ.

[24] Postma TC, White JG (2016). Developing integrated clinical reasoning competencies in dental students using scaffolded case-based learning - empirical evidence. Eur J Dent Educ.

[25] Dennis MJ, Bennett JD, DeLuke DM, Evans EW, Hudson JW, Nattestad A (2017). Improving the medical curriculum in predoctoral dental education: recommendations from the american association of oral and maxillofacial surgeons committee on predoctoral education and training. J Oral Maxillofac Surg.

[26] Emam HA, Jatana CA, Wade S, Hamamoto D (2018). Dental student self-assessment of a medical history competency developed by oral and maxillofacial surgery faculty. Eur J Dent Educ.

[27] Dinsbach NA (2012). Antibiotics in dentistry: bacteremia, antibiotic prophylaxis, and antibiotic misuse. Gen Dent.

[28] Bahammam MA, Abdelaziz NM (2015). Awareness of antimicrobial prophylaxis for infective endocarditis among dental students and interns at a teaching hospital in Jeddah. Saudi Arabia Open Dent J.

